# Managing a Tertiary Orthopedic Hospital during the COVID-19 Epidemic, Main Challenges and Solutions Adopted

**DOI:** 10.3390/ijerph17134818

**Published:** 2020-07-04

**Authors:** Francesco Magro, Paolo Perazzo, Elena Bottinelli, Francesco Possenti, Giuseppe Banfi

**Affiliations:** 1 IRCCS Orthopedic Institute Galeazzi, 20161 Milan, Italy; paoloperazzo1@virgilio.it (P.P.); elena.bottinelli@grupposandonato.it (E.B.); francesco.possenti@grupposandonato.it (F.P.); banfi.giuseppe@hsr.it (G.B.); 2 Vita-Salute San Raffaele University, 20132 Milan, Italy

**Keywords:** access facility, clinical pathways, COVID-19, organization and administration, hospital, workforce

## Abstract

The present paper is a review of the main challenges faced by the management of a tertiary specialty hospital during the COVID-19 pandemic in the northern Italian region of Lombardy, an area of extremely high epidemic impact. The article focuses on the management of patient flows, access to the hospital, maintaining and reallocating staffing levels, and managing urgent referrals, information, and communications from the point of view of the hospital managers over a seven-week period. The objective of the article is to provide beneficial insights and solutions to other hospital managers and medical directors who should find themselves in the same or a similar situation. In such an epidemic emergency, in the authors’ opinion, the most important factors influencing the capability of the hospital to maintain operations are (1) sustaining the strict triage of patients, (2) the differentiation of flows and pathways to create what could be regarded as “a hospital inside a hospital”, (3) tracing and sharing all available information to face the rapidly changing environment, (4) being able to maintain staffing levels in critical areas by flexibly allocating the workforce, and (5) from a regional perspective, being organized along a hub-and-spoke system for critical and time-sensitive networks was key for focusing the hospital’s resources on the most needed services.

## 1. Background

Istituto Ortopedico Galeazzi is a 364 bed multispecialty private orthopedic hospital in Milan, in the northern Italian region of Lombardy, which delivers care to the public on behalf of the national payor. Galeazzi performs about 17,000 surgical operations every year, and is also the hospital which performed the highest number joint replacement surgeries in the country (approximately 4000 in 2019) and performs over 1600 complex spine surgeries per year. The hospital is a primary emergency center for isolated orthopedic trauma.

In the months of February and March 2020, the northern Italian region of Lombardy was the most severely impacted region of the Western world by the COVID-19 pandemic [1,2]. As widely reported, the epidemic had a major impact on the provision and organization of healthcare services throughout the region of Lombardy [3]. While Galeazzi was previously a hospital mostly dedicated to elective orthopedic surgical cases (93% of admissions were elective and only 7% were urgent admissions between February and April of 2019) (Table 1) at the height of the epidemic, the hospital became dedicated exclusively to:

(a) the admission of medical COVID-19 patients overflowing from the surrounding general hospitals, admitted to Galeazzi both on ordinary wards and the intensive care unit;
(b) urgent trauma admissions and urgent-elective cases which could not be dealt with by other specialty hospitals because their own orthopedic wards were replaced by COVID-19 wards.

During the seven weeks covered by this report, the hospital’s rate of urgent admissions rose from 7% to approximately 30% during the whole period, with approximately 80% of admissions being urgent in nature in the last three weeks of the period [4] (Table 2).

Over the same period, outpatient episodes were reduced by approximately 90% due to the regulator′s request to cancel all non-urgent activity.

The timeline of events begins on Friday, 21 February, when the regional authorities of Lombardy declared the so-called “red zone” over 10 municipalities of the region, blocking entry or exit from these municipalities, and mandating hospitals to take the first countermeasures. The events are contained in a seven-week period ranging from Monday 24 February to Sunday 12 April 2020, the day on which this article was started, encompassing the peak of the epidemic phase in the region [5].

## 2. Managing Access to the Hospital

The first indication to restrict access to the hospital came from the regional authorities on February 21 when all hospitals of the region were requested to cancel all appointments and admissions of patients residing in the so-called “red zone”. Later in the same week (WK1), Galeazzi management spontaneously restricted all access to clinical auditors and observers, product specialists, and students, whose presence was not essential for clinical activities. During the first week, Galeazzi’s approach was to perform a clinical history check for all elective admissions at the time of the administrative admission in the admissions office. “Suspected cases” were identified as follows: individuals who reported temperature rises, influenza-like symptoms and a history of travel to mainland China or “red zones”, or of exposure to a known COVID-19 case in the previous two weeks. These individuals were refused access to the wards and other clinical areas. The choice to adopt the two elements (symptoms + travel or traceable exposure) as criteria for access restriction was based on the first definition issued by the Italian Ministry of Health of a “suspected case”, and was later expanded during week 2 to only include the symptoms, regardless of history of travel or known exposure [6].

During the second week, in response to a rapid progression of the epidemic, the hospital modified its access policy heavily compared to the previous week. Whilst in the first week, all access doors to the hospitals were open and temperature and clinical history checks on patients and visitors were performed only before accessing clinical treatment areas, during the second week, all entrances to the hospital building were blocked, with the exception of the main entrance and the ER. A screening area was set up at the entrance of the hospital in the main lobby with three to five nurses during every shift performing a temperature check on every patient and visitor, and collecting clinical information regarding influenza-like symptoms and recent travel. Visitors or patients identified as “suspected cases” were turned away and asked to rebook the appointment at least two weeks after the symptoms had disappeared. “Suspected cases”, who were attending the hospital for essential services (e.g., cast removals), were equipped with surgical masks and gloves and allowed to attend their appointments. Nurses performing the checks were equipped with a surgical facial piece, a plastic transparent visor protecting the face, gloves, and a single-use full body gown (non-sterile). In the emergency room, the triage room was isolated from the rest of the treatment areas and a nurse with full COVID-19 grade (category III bio-hazard protection) PPE performed a clinical examination for temperature and other symptoms suggestive of COVID-19. Limiting access to the hospital to only two points allowed the hospital to exert complete control on the number of people entering and block ones with clinical symptoms suggestive of COVID-19. Additional limitations to public access were put in place, as follows:

- Access to relatives accompanying patients to their outpatient appointments was forbidden from the middle of week 2 onwards.
- Inpatient visitors were limited to only one per day per patient from the beginning of week 3, and visiting hours were heavily reduced.
- All visits to patients in the hospital were completely canceled from the end of week 3 onwards.

## 3. Clinical Workforce Management

The COVID-19 epidemic placed a particular strain on the human resources of the hospital in many ways and we identify what we believe to be the three main ways in which this strain was exerted.

Lack of staff—Under normal circumstances, the hospital can count on 687 employed staff, of which 540 work in a clinical capacity and 147 in a non-clinical capacity.

The onset of the emergency put the hospital’s HR under extreme pressure due to the limited availability of clinical resources (medical, nursing and healthcare assistants, as well as clinical technicians). Clinical staff were the most hard hit by epidemic-related absences. Epidemic-related absences included different cases:

- Employees who were themselves unwell, either due to COVID-19 or other forms of illness.
- Employees exempted from work by the hospital’s occupational doctor due to permanent medical conditions. This included, for example, workers on immunosuppressive therapy or with severe pre-existing conditions who could not take the risk of being exposed to COVID-19.
- Employees who had to care for sick relatives.
- Employees living in social circumstances incompatible with epidemic exposure. For example, ones who lived with especially frail relatives and did not have any possibility to isolate themselves.

**Figure 1:**
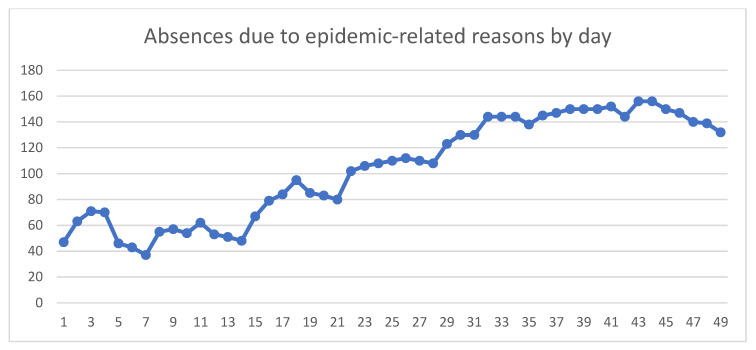
Employees on leave by day, for reasons related to the epidemic between 24 February 2020 and 12 April 2020.

On 24 February, people on epidemic-related absence amounted to 47. At the beginning of week 4 on Monday the 16th of March, 102 people were absent due to epidemic-related reasons, and on April 2nd (WK6), the number of staff on epidemic-related absence peaked at 156, of which there were seven physicians, 72 nurses, 19 administrative staff, 15 lab/rad technicians, and 43 healthcare assistants. The 156 absent staff members represented 22.7% of the hospital’s total employed staff, and were mostly patient-caring staff (137 people, or 87.8% of the total absent staff) (Figure 1).

Another 250 people, mostly physicians, however, were employed on the hospital’s flexible freelance contracts, which do not require reporting days of absence. While several of these people did not attend the hospital on multiple working days, it is impossible for the hospital’s administration to know how many of these absences were related to the ongoing epidemic.

To cope with these severe shortages of nurses and healthcare assistants, the overall number of staffed beds was reduced and occupied beds were concentrated in fewer wards to ensure a maximum ratio of staff/patients. Seven of the hospital’s 15 wards/units were closed, totaling 150 beds due to logistic reasons (the need to use wards that could be physically separated from the rest of the hospital to avoid disease spread) and staff availability, and the hospital continued to operate with the remainder of beds.

Psychological and physical stress—Galeazzi normally has a group of psychologists available for staff to provide support with work-related stress topics. Under normal circumstances, employees can connect with these psychologists, who provide free support during two initial consultations and further free consultations to the employee if the cause of the psychological stress is identified as being work related. During the epidemic, the psychologists reached out proactively to staff and made themselves available for consultations, especially to staff working in COVID-19 units. Due to the ongoing epidemic, the psychologists offered consultations with the staff via videoconferencing to avoid physical proximity.

Physical fatigue was another major issue of staff working in COVID-19 units. Long hours and the PPE that the staff must wear if working with COVID-19 patients are extremely demanding on the body. Previously, nursing and healthcare assistants’ shifts on wards followed a 7-7-10 hourly structure (seven hours for the morning shift, seven hours for the afternoon shift, and 10 h for the night shift). To avoid extreme fatigue, all shifts of the hospital for clinical staff were changed to 6-6-6-6 h over 24 h. While the previous shift structure took five days of work a week to fulfill the 36-hour weekly amount, the new structure required six days of work a week. This brought mainly two types of benefits. First of all, the shorter shift was physically better tolerated by staff. Secondly, the fact that staff wearing full body PPE often chose to avoid bathroom, feeding, and drinking breaks until the end of the shift (which would have entailed a complete change/shower and the usage of a second set of equipment per shift) led to a decrease in the overall usage of PPE.

Workforce reallocation—The emergency completely subverted the usual case mix of the hospital. Galeazzi is mainly an orthopedic center, however, it holds numerous other specialties. Many of the specialties that were normally active before the emergency were suspended because they were either identified as noncritical or assigned to other hospitals in the healthcare network. Given the radical change in the case mix, all of the medical staff who could not be used in their normal function were assigned to other functions, mainly in the COVID-19 units. COVID-19 medical wards were staffed around the clock by a team of four physiatrists, four cardiologists, two neurosurgeons, two neurologists, two dermatologists, one vascular surgeon, two maxillofacial surgeons, two pain therapists, and five to seven orthopedic surgeons. These were employees of the hospital, as well as doctors holding a freelance contract with the hospital who volunteered to do shifts on the COVID-19 units. Anesthesiologists/intensivists were mostly staffed in intensive care units or surgical theaters, however, one anesthesiologist was based on the COVID-19 wards 24 h a day to assist other specialists in critical situations for both medical and surgical COVID-19 patients (e.g., placement of CPAP, central venous access, decompensations, and transfers to intensive care).

## 4. Managing Patient Flows

The proactive and well-informed management of patient flows with very strict separation between COVID-19 and non-COVID-19 was the single most important element in ensuring the safety of patients and operators and maintaining the hospital activity. The key learning point for Galeazzi during this emergency is that the hospital must be able to create two different hospitals out of one, with separate paths for COVID-19 and non-COVID-19 patients operating independently.

Galeazzi went through two different phases of the operational management of patient flows. While the structure and direction of flows remained the same as described below, the only difference between the two different phases was the mode of reconnaissance of COVID-19 cases. In weeks 1–4, patients were assigned to COVID-19 or non-COVID-19 units based on the presentation of clinical symptoms within 72 h of admission on the “quarantine” ward, without systematically performing a rhino-pharyngeal swab at the time of admission. Only patients displaying COVID-19 symptoms were swabbed, in accordance with the regional public health authority guidance. In weeks 3–7 and to date, every single patient is swabbed at the time of admission on the quarantine ward and is subsequently transferred to the COVID-19 or non-COVID-19 units based on their result within the first 24 h. In hindsight, systematically swabbing every patient upon admission is the advisable mode of action compared to awaiting the display of clinical symptoms. It has, in fact, since been reported that COVID-19 can be transmitted by non-symptomatic patients and can be transmitted before the onset of symptoms.

### 4.1. ER

The emergency room was chosen as the only mode of access for new admissions to the hospital. Identified COVID-19 cases transferred to Galeazzi from other hospitals are transported directly to COVID-19 units through a direct access elevator next to the ER used exclusively for the transportation of infected cases without entering the ER rooms. For other cases of planned orthopedic surgery transfers and trauma cases, the access to the ER happens through an isolation triage room with a nurse in full COVID-19 grade PPE, who assesses the presence of signs and symptoms of COVID-19, as well as performing the ER triage. All patients with an unclear COVID-19 status (excluding patients arriving with a negative swab performed within the last 24 h) are placed into two isolation rooms in the ER. After the patients clears the ER, all of the physical spaces in which the patient has transited, including rooms, hallways, and elevators are sanitized by means of a peroxide aerosol and sodium hypochlorite wipes for surfaces.

### 4.2. Admission Units

All patients entering the hospital are initially admitted to a dedicated “quarantine” ward on the hospital’s third floor, where a rhino-pharyngeal swab for COVID-19 diagnosis is performed immediately. The ward is managed with the complete isolation of the patients from each other and all of the staff accessing the ward are in full COVID-19 grade PPE. The patients remain admitted to the quarantine ward until the results of the swab are obtained, usually less than 24 h from the time it is performed.

Patients with a negative swab are subsequently transferred to the “clean” wards on the hospital’s fourth and sixth floors. Patients with a positive swab are transferred to the COVID-19 units on the hospital’s second floor.

### 4.3. Operating Rooms

Since the beginning of the COVID-19 emergency, Galeazzi has decreased the number of active operating theaters. Under normal circumstances, the hospital operates with 11 operating theaters on two different floors open from 7:30 a.m. to 7:30 p.m. Monday to Friday, and five operating theaters on one single floor open for a half-day on Saturday. The hospital also has three small ORs for ambulatory surgery which are used Monday–Friday. While the during the first two weeks, emergency elective surgery was preserved at least partially, from the third week onwards, all elective non-urgent surgery was completely stopped. The need to operate on trauma cases with a positive or unknown COVID-19 status was initially managed by operating on COVID-19 cases in non-dedicated operating theaters as the last cases of the day. The patients were brought into the surgical area after all other patients had been cleared, managed by staff with COVID-19 grade PPE and the whole area was sanitized by peroxide aerosol and active chlorine in sodium hypochlorite for surfaces after the end of the surgery. During week three, however, the hospital began technical works (the placement of a temporary wall and the separation of ventilation) to functionally split the remaining active surgical floor into two separate areas, one for COVID-19 surgery (two rooms) and the other for non-COVID-19 cases (three rooms). From the beginning of week four, the two areas were completely separate from each other, with independent pathways and access for patients, as well as for staff. The COVID-19 theaters were also connected directly through an elevator to the COVID-19 ICU in the hospital. Only patients with a negative rhino-pharyngeal swab could access the non- COVID-19 theaters, while all patients with a positive swab or without a swab result were treated by a dedicated trauma team wearing full COVID-19 grade PPE in the COVID-19 section. Having a single COVID-19 surgical team work throughout the day on COVID-19 cases allowed the hospital to maximize efficiency (changing time was saved) and save precious PPE.

### 4.4. Intensive Care Units

Under normal circumstances, Galeazzi has eight active ICU beds, of which only six are active and used exclusively for postoperative stays. At the beginning of week 3, the extreme need for ICU beds led Galeazzi to activate two different ICUs, for COVID-19 and non-COVID-19 patients.

In week 3, the main ICU of the hospital, with four beds, was dedicated to COVID-19 cases, while an additional four ICU beds were obtained from one of the surgical theaters’ recovery areas for non-COVID-19 patients. In week 4, the COVID-19 ICU was moved into the surgical area of the fifth floor (all six operating rooms of this this surgical floor were inactivated due to the decrease in elective surgery and were entirely converted into a COVID-19 ICU) where it currently remains at the time of the writing of this article. The maximum occupancy of the COVID-19 ICU was 10 contemporary patients during week 6.

## 5. Management of Urgent Referral Network Response

As mentioned at the beginning of this paper, the COVID-19 emergency determined a complete reorganization of the urgent referral network at a regional level. The regional healthcare authorities identified a number of COVID-19 hospitals that were mainly dedicated to the treatment of COVID-19 cases and would not be able to deal with the normal caseload in other specialties, as they did during normal times. On 14 March (end of week 3), a regional decree (Welfare Directorate Decree n. 3351 of the Region of Lombardy) was issued, which designated the hub and spoke hospitals for all time-sensitive networks in all specialties [7,8,9]. Galeazzi was one of the two hospitals designated to deal with all urgent and urgent-planned orthopedic cases for the metropolitan area of Milan. This is a catchment area of approximately 5 million inhabitants divided among two hospitals. Patients therefore reached the hospital from two main sources: the territorial ambulance network and transfers from other hospitals who were not allowed to treat their orthopedic cases because of their designation within the new organization. This determined an increase in trauma cases requiring admission and early surgery (within 48 h from admission) from approximately two to three cases per day to 7–12 per day. The two peak days during weeks five and six had 12 surgical admissions per day.

The new demand for urgent surgery exceeded the capacity of the Orthopedic Trauma team, which in Galeazzi’s organization is in charge of staffing the ER, performing trauma surgery, and staffing trauma outpatient clinics.

In response to the new situation, the hospital’s Operations Department restructured the shift organization for all urgent services in the hospital using staff from other orthopedic teams normally designated to elective cases, instead of just the trauma team.

- daytime ER shifts were distributed among all orthopedic teams;
- two additional on-call shifts were added to cover the day so that the medical presence in the ER could be doubled upon request when needed;
- senior surgical coverage overnight (on call, not in person) normally provided by five trauma surgeons was doubled by adding five senior orthopedic surgeons from other teams with trauma experience;
- an additional surgical team was made available every day to treat femur fractures, staffed by three other surgical teams experienced in hip surgery, on a rotational basis;
- three trauma outpatient clinics used for surgical follow-ups for trauma surgery were doubled using surgeons from other teams.

## 6. Information and Communications Management

Managing information and ensuring that everybody was always “on the same page” in the rapidly changing environment of the emergency was a critical factor throughout the emergency. Galeazzi adopted the below solutions to ensure this was achieved in different stages of the emergency.

From the very beginning of the emergency (on 21st February), an instant messaging group was created on a widespread mobile application to share important information with all the main stakeholders in the hospital. Before other, more structured, methods of sharing information were established in the hospital, this allowed members of staff to quickly communicate with all the involved people at the same time. Despite this being a rather unorthodox solution, it proved extremely effective in achieving the goal of the instant transfer of information among the management team.

This group included the chief executive officer, chief operating officer, medical director, the staff of the medical directorate, the head nurse, the IT manager, the head of technical services, the manager of clinical engineering, the head of laboratory services, the risk manager, the head of the health and safety department, the head of anesthesiology and intensive care, the head of trauma surgery, and the manager of patient services. Subsequently, when COVID-19 units were created, the physician leads of the COVID-19 medical and surgical wards, as well as the physician lead for the non-COVID-19 wards, were added.

Subsequently, medium-range portable radio transmitters were used to coordinate ongoing activities, such as admissions, patient transfers, or other situations requiring real-time management. Radios were provided to the chief operating officer, the head of nursing, the head of technical services, the physician head of anesthesia and intensive care, the risk manager, the nurse coordinators of the ER, operating rooms, and COVID-19 intensive care, the lead physician of the COVID-19 unit, and to the on-call person of the medical directorate. All holders stayed on stand-by on the same channel so that all staff provided with radio could listen and be aware of the contents of the interactions.

Other, more structured changes, were made to the hospital’s IT system. A real-time tracing system of the results of rhino-pharyngeal swabs for all admitted patients was created on the hospital’s principal planning software. Given the paramount importance of being able to separate patient flows between COVID-19 and non-COVID-19, this allowed all staff to instantly verify the COVID-19 status of a patient, and which paths the patient should follow. Whichever staff member performed the swab added the new swab on the system, which appeared as pending result. Subsequently, when the results were acquired by the hospital’s lab system, this integrated automatically with the software, showing the swab result in real time. This was particularly important in the first 24 h of the patient’s admission in the hospital because it allowed staff to determine subsequent movements in terms of the OR to be used (COVID-19 vs. non-COVID-19) and destination wards for new admissions.

Another add-on to the hospitals OR management software was quickly implemented to trace urgent admissions requiring surgery. This feature allowed to staff to visualize and locate all urgently admitted patients in need of surgery and their status with regards to surgery (e.g., in preparation, in OR, operated, etc.). This was especially useful for the anesthesia and surgical teams to track and trace newly admitted patients to be prepared for surgery.

## 7. Conclusions

The COVID-19 emergency was an extremely difficult period for Galeazzi and for the whole healthcare system of Lombardy. Many clinical departments from multiple hospitals, as well as healthcare regulators and organizations, reported similar difficulties to the ones experienced by Galeazzi [4,5,6,7,8,9]. All departments of the hospital, both clinical and nonclinical, were severely strained to an extent which could not have been imagined previously. In the authors’ experience, the most important lessons learned and the critical factors to maintain operations during the emergency were the following:

(1) Strictly triaging patients is paramount. The detection of COVID-19 cases no later than at the time of admission to the hospital is required to maintain the safety of patients and staff. Undiagnosed patients must be treated as potentially affected until proven otherwise.
(2) The hospital must be able to function as two separate hospitals, a COVID-19 hospital and a non-COVID-19 hospital, with clearly identified pathways that never meet.
(3) An “all hands on deck” approach must be used throughout the emergency, and all of the critical stakeholders must be “on the same page” at all times to ensure a coordinated reaction to the changing environment. Instantaneous means of communication (high speed, wide reach) must be preferred.
(4) Every effort must be made to preserve staffing levels, this requires reallocating staff flexibly to different areas of the hospital according to need and availability regardless of previous allocations.
(5) On a regional perspective, being organized along a hub-and-spoke system for critical and time-sensitive networks was key to focusing the hospital’s resources on the most needed services, while more general hospitals coped with the bulk of COVID-19 patients.

## Figures and Tables

**Table 1:** Planned vs. urgent acute admissions between 24 February 2019 and 12 April 2019, by week.

Type of Admission	Week 1	Week 2	Week 3	Week 4	Week 5	Week 6	Week 7
Planned	308	290	328	286	313	332	138
Urgent *	24	26	21	27	18	20	9
% urgent cases	7.2%	8.2%	6.0%	8.6%	5.4%	5.7%	6.1%

* Includes ambulance emergency network admissions, ER admissions and transfers from other hospitals.

**Table 2:** Planned vs. urgent acute admissions between Feb 24th and April 12th 2020, by week.

Type of Admission	Week 1	Week 2	Week 3	Week 4	Week 5	Week 6	Week 7
Planned	158	282	147	39	15	12	11
Urgent *	26	23	42	38	51	52	36
% urgent Cases	14.1%	7.5%	22.2%	49.4%	77.3%	81.3%	76.6%

* Includes ambulance emergency network admissions, ER admissions and transfers from other hospitals.
